# S100A2 activation promotes interstitial fibrosis in kidneys by FoxO1-mediated epithelial-mesenchymal transition

**DOI:** 10.1007/s10565-024-09929-7

**Published:** 2024-10-09

**Authors:** Xuejia Yang, Fan Zheng, Penghua Yan, Xueting Liu, Xuanwen Chen, Xinyu Du, Yin Zhang, Peilei Wang, Chaosheng Chen, Hong Lu, Yongheng Bai

**Affiliations:** 1 Zhejiang Key Laboratory of Intelligent Cancer Biomarker Discovery and Translation, The First Affiliated Hospital of Wenzhou Medical University, Wenzhou, 325035 China; 2 Department of Laboratory Medicine, The First Affiliated Hospital of Wenzhou Medical University, Wenzhou, 325035 China; 3 Department of Nephrology, The First Affiliated Hospital of Wenzhou Medical University, Wenzhou, 325035 China; 4 Department of Pathology, The First Affiliated Hospital of Wenzhou Medical University, Wenzhou, 325035 China; 5 Institute of Chronic Nephropathy, Wenzhou Medical University, Wenzhou, 325035 China

**Keywords:** RIF, S100A2, FoxO1, EMT, ECM

## Abstract

**Background:**

Renal interstitial fibrosis (RIF) is a common feature of chronic kidney diseases (CKD), with epithelial-mesenchymal transition (EMT) being one of its important mechanisms. S100A2 is a protein associated with cell proliferation and differentiation, but its specific functions and molecular mechanisms in RIF remain to be determined.

**Methods:**

S100A2 levels were evaluated in three mouse models, including unilateral ureteral obstruction (UUO), ischemia-reperfusion injury (IRI), and aristolochic acid nephropathy (AAN), as well as in TGF-β1- treated HK-2 cells and in kidney tissue samples. Furthermore, the role of S100A2 and its interaction with FoxO1 was investigated using RT-qPCR, immunoblotting, immunofluorescence staining, co-immunoprecipitation (Co-IP), transcriptome sequencing, and gain- or loss-of-function approaches *in vitro*.

**Results:**

Elevated expression levels of S100A2 were observed in three mouse models and TGF-β1-treated HK2 cells, as well as in kidney tissue samples. Following siRNA silencing of S100A2, exposure to TGF-β1 in cultured HK-2 cells suppressed EMT process and extracellular matrix (ECM) accumulation. Conversely, Overexpression of S100A2 induced EMT and ECM deposition. Notably, we identified that S100A2-mediated EMT depends on FoxO1. Immunofluorescence staining indicated that S100A2 and FoxO1 colocalized in the nucleus and cytoplasm, and their interaction was verified in Co-IP assay. S100A2 knockdown decreased TGF-β1-induced phosphorylation of FoxO1 and increased its protein expression, whereas S100A2 overexpression hampered FoxO1 activation. Furthermore, pharmacological blockade of FoxO1 rescued the induction of TGF-β1 on EMT and ECM deposition in S100A2 siRNA-treated cells.

**Conclusion:**

S100A2 activation exacerbates interstitial fibrosis in kidneys by facilitating FoxO1-mediated EMT.

**Graphical abstract:**

A schematic diagram of the underlying mechanisms by which S100A2 regulates EMT and renal fibrosis. Following injury, the cytoplasmic expression of S100A2 in renal tubular epithelial cells is markedly elevated. This increase promotes the phosphorylation of FoxO1, preventing its translocation into the nucleus and enhances EMT and extracellular matrix ECM deposition, thereby exacerbating renal interstitial fibrosis. 
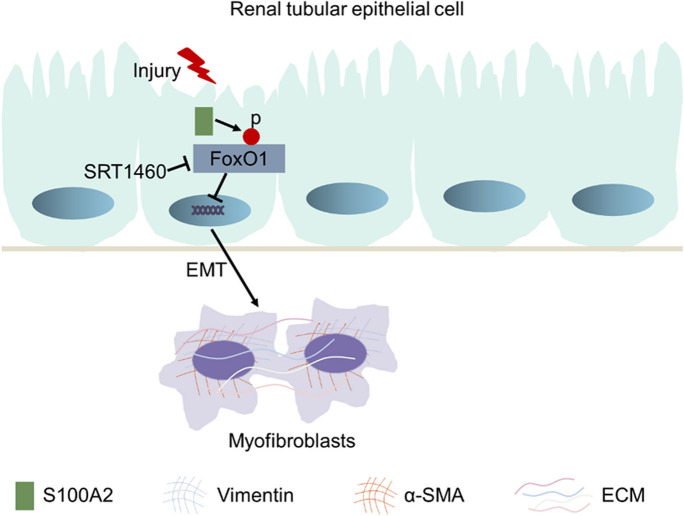

**Supplementary Information:**

The online version contains supplementary material available at 10.1007/s10565-024-09929-7.

## Introduction

Renal interstitial fibrosis (RIF) is the ultimate manifestation of various forms of chronic kidney disease (CKD), resulting in progressive irreversible damage to kidney function and posing a significant threat to human health (Humphreys 2018). Evidence suggests that CKD impacts 10% to 14% of people worldwide. RIF is a complex dynamic process characterized by excessive accumulation and deposition of extracellular matrix (ECM), leading to the formation of fibrotic scars, organ parenchymal destruction, and functional loss (Lovisa et al. 2016). Despite ongoing efforts to identify safe and effective strategies for anti-fibrotic therapy (Cha et al. 2020; Liu et al. 2021; Vanhove et al. 2017; Wu 2019), there is currently no definitive treatment for RIF. Elucidating the complex cellular and molecular mechanisms of RIF is of paramount importance for the development of effective therapeutic strategies to cure or delay the loss of kidney function. During the development of RIF, proximal tubular epithelial cells (PTECs) are considered the most sensitive cells to injury (Chevalier 2016). Under the influence of injurious factors, PTECs respond to injury by reorganizing the cytoskeleton of actin filaments and expressing de novo markers of mesenchymal cells, while still retaining some epithelial characteristics, a process known as partial epithelial-to-mesenchymal transition (EMT) (Bozic et al. 2020). Although phenotypically transformed PTECs cannot traverse the basement membrane, they can further promote inflammation and induce RIF and tissue damage through paracrine signaling (Grande et al. 2015). Therefore, the phenotypic transformation of PTECs plays a crucial role in driving the formation of RIF.

S100A2 is one of the members of the S100 protein family, playing important roles in human physiology and pathology by regulating the activity of downstream target proteins. The S100 protein family comprises over 20 members with unique structural features, including two EF-hand calcium-binding domains separated by a hinge region, with an amino-terminal (N-) and carboxy-terminal (C-) end. The regular C-terminal EF-hand exhibits a 100-fold higher affinity for calcium ions than the irregular N-terminal EF-hand. The C-terminal region varies among different S100 proteins, potentially providing selective binding sites for specific target proteins (Doi et al. 2021). The S100A2 gene spans 8,670 base pairs and is located on human chromosome 1q21 between 250 and 300 kilobases, consisting of three exons and two introns, encoding a 97-amino acid S100A2 protein. So far, extensive research has demonstrated the involvement of S100A2 in various aspects of tumor proliferation, invasion, EMT, and prognosis prediction (Chen et al. 2023b; Chen et al. 2021; Hatthakarnkul et al. 2023; Wang et al. 2021). However, the connection between S100A2 and RIF and its specific mechanisms of action remain unclear.

Several transcription factors and signaling pathways participate in the EMT process, such as FoxO1, TGF-β/smad2/3, and Wnt/β-catenin signaling pathways (Lai et al. 2021; Tian et al. 2023; Wang et al. 2023). FoxO1 signaling can function both as a transcription factor regulating target gene activity and as a target regulated by other molecules. Studies have found that *in vivo* lentivirus-mediated overexpression of FoxO1 inhibited EMT in podocytes of diabetic mice by suppressing TGF-β1/Smad3/ILK, significantly alleviating diabetes-induced renal injury and proteinuria, consistent with *in vitro* cell experimental results (Du et al. 2016). STYK1 enhances the migration, invasion, and EMT of non-small cell lung cancer by inhibiting FoxO1 signaling, increasing the expression of transcription factors Snail1 and zinc finger E-box-binding homeobox 2 (Zeb2), and mesenchymal marker vimentin, and reducing the levels of epithelial marker E-cadherin (Lai et al. 2021). However, whether there is an interaction between S100A2 and FoxO1 and their mechanisms of action require further investigation.

In this study, we established three RIF models in mice and assessed the expression and localization of S100A2. Clinical pathological samples were also utilized to evaluate S100A2 expression in RIF. Subsequently, we investigated the specific mechanisms of S100A2. Our findings indicated that S100A2 promotes EMT by inhibiting FoxO1, a process effectively blocked by the FoxO1 inhibitor SRT1640. This suggests a potential regulatory role of S100A2 in EMT through FoxO1, thereby influencing RIF progression. These results highlight S100A2 as a promising target for the prevention and treatment of RIF, as well as for the development of novel therapeutic interventions.

## Materials and methods

### Animal care and model construction

As previously described (Zhang et al. 2019), male C57BL/6 mice, aged 6-8 weeks, were purchased from the Experimental Animal Center of the First Affiliated Hospital of Wenzhou Medical University. The mice were housed under conditions of 22-25°C temperature, 40-60% humidity, and a 12-hour light/dark cycle, provided with standard chow and water. The mice were divided into four groups: sham (UUO7d and UUO14d) and unilateral ureteral obstruction (UUO) groups (UUO7d and UUO14d), with 6 mice per group. Under anesthesia with isoflurane, mice in the UUO groups underwent a midline incision to expose and ligate the left ureter, inducing obstruction, followed by suturing of the incision. The sham group underwent exposure of the left kidney without ureteral ligation. Mice were euthanized with isoflurane anesthesia at 7 and 14 days post-surgery, and kidneys were harvested for histological analysis. For the ischemia-reperfusion injury (IRI) model, an equal number of mice were randomly allocated to four groups: sham (IRI24h and IRI72h) and IRI groups (IRI24h and IRI72h). After administering anesthesia with isoflurane, two small incisions were made on the dorsal side to expose the kidneys. A non-traumatic microvascular clamp was used to occlude both renal pedicles for 40 minutes, blocking blood flow to the kidneys.

Following the ischemic period, the clamps were removed. Mice were sacrificed at 24 hours and 72 hours after reperfusion, and the kidneys were harvested for further analysis (Chen et al. 2023a). For the study of Aristolochic Acid Nephropathy (AAN), mice were randomly divided into two groups: the control group and the AAN group. Mice in the AAN group were administered Aristolochic Acid (Sigma-Aldrich, 3 mg/kg dissolved in PBS) via intraperitoneal injection every other day for 3 weeks, followed by a 3-week recovery period. Mice injected with PBS served as the control group. All mice were euthanized with isoflurane after 6 weeks of injections.

### Clinical specimen collection

A total of 80 renal pathology slides from patients with IgA nephropathy, diabetic nephropathy, hypertensive nephropathy and lupus nephropathy in the First Hospital of Wenzhou Medical University were collected for the study, and they were classified into low-fibrosis (*n*=10) and high-fibrosis (*n*=10) groups according to the degree of fibrosis.

### Histopathological examination

The renal specimens were preserved using 4% paraformaldehyde, embedded in paraffin, and sliced into 4 μm thick sections. According to the manufacturer's instructions, histological assessment of renal injury and fibrosis was performed on paraffin sections of mouse kidney tissue using Hematoxylin and Eosin (HE) staining (Solarbio, China) and Masson's trichrome staining (Solarbio, China). Twenty non-overlapping regions were randomly selected and imaged for each mouse. The severity of renal injury was assessed based on the intensity of tubular dilation (<25%, 25%-50%, 50%-75%, and 75%-100%, scored as 1, 2, 3, and 4, respectively). ImageJ software was utilized for quantitative analysis of renal fibrosis, expressed as the average percentage of fibrotic area relative to the total area.

### Immunohistochemistry (IHC) staining

The kidney tissues were initially treated with xylene to remove wax and then progressively rehydrated through ethanol solutions (100%, 95%, 85%, and 75%), concluding with distilled water, followed by treatment with 3% hydrogen peroxide. Subsequently, the sections were blocked with goat serum for one hour, after which they were incubated with primary antibodies for 12-16 hours at 4°C, including anti-E-cadherin (Abcam, USA), N-cadherin (Abcam), type I collagen (Proteintech, USA), Vimentin (Proteintech), α-SMA (Santa Cruz, USA), and S100A2 (Abcam). Following PBS washing, HRP-labeled secondary antibodies were applied, and DAB chromogenic staining was performed, followed by hematoxylin counterstaining. Imaging was carried out using a Leica microscope.

### Cell culture and treatment

Human kidney proximal tubular cells (HK-2), obtained from the Cell Bank of the Chinese Academy of Sciences (Shanghai, China), were maintained in DMEM/DF-12 medium (Gibco, USA) enriched with 10% fetal bovine serum and 1% penicillin-streptomycin. The HK-2 cells were treated with TGF-β1 (20 ng/mL) for 48 hours to induce fibrosis-like changes. The cells were also exposed to 200 ng/ml of recombinant S100A2 protein (MCE, USA) or 50 μM SRT1460 (MCE) for 24 hours.

In this study, HK2 cells were treated with TGFβ1-IN-1 (HY-151427, MCE), a specific TGF-β1 inhibitor, at a concentration of 20 μM for 24 hours to evaluate its efficacy in blocking the effects of TGF-β1 on S100A2 expression. Additionally, to simulate hypoxia-induced renal injury, HK2 cells were exposed to 500 μM CoCl2 for 24 hours, and the subsequent changes in S100A2 expression were assessed before and after treatment.

### Cell transfection

S100A2-siRNA was sourced from GenePharma (China). Prior to transfection, the cell culture medium was substituted with Opti-MEM (Invitrogen). Upon reaching approximately 70% cell density, transfection was conducted utilizing Lipofectamine 2000 (Invitrogen), following the manufacturer's protocol. The siRNA (50 μM) and Lipofectamine 2000 (5 μl) were separately mixed with Opti-MEM (250 μl). After a 15-minute incubation period, the diluted solutions of siRNA (250 μl) and Lipofectamine 2000 (250 μl) were combined and introduced to HK-2 cells for incubation. Following 24 hours, the medium was replaced with 10% FBS medium. Subsequently, TGF-β1 (20 ng/mL) was introduced to the HK-2 cells and allowed to incubate for an additional 24 or 48 hours prior to RNA or protein extraction.

### RT-qPCR

Total RNA was isolated from kidney tissues and HK-2 cells using Trizol reagent (Glpbio, USA). Subsequently, the RNA was reverse transcribed to generate cDNA using the HiScript III 1st Strand cDNA Synthesis Kit (Vazyme, China). Quantitative PCR experiments were conducted using SYBR Green reagent (Vazyme), with β-actin serving as an endogenous reference gene. For quantitative analysis, all samples were assessed using the 2^-ΔΔCt^ method. The relevant primer sequences are provided in Supplementary Table 1.

### Immunoblotting and Co-inmunoprecipitation (Co-IP) experiments

Kidney tissue and HK-2 cell proteins were isolated and separated using SDS-PAGE gels and subsequently transferred onto a PVDF membrane. After blocking with 5% non-fat milk, the membrane was probed with the relevant primary antibodies overnight including anti-Vimentin (Santa Cruz), E-cadherin (Abcam), α-SMA (Santa Cruz), N-cadherin (Abcam), COL1 (Proteintech), S100A2 (Abcam), Smad2/3 (Abcam), p-Smad2/3 (Thr8, Abcam), p-PI3K (Tyr607, Affinity, USA), PI3K (Proteintech), p-AKT (Ser473, CST), AKT (CST), Sirt1 (Affinity), p-FoxO1 (Ser256, Affinity), FoxO1 (Affinity), Snail1 (Abcam), COL3 (Proteintech), GAPDH (Proteintech), H3 (Proteintech), and β-actin (Affinity). On the following day, the membrane was washed and incubated with the corresponding HRP-labeled secondary antibody. Chemiluminescence was then developed, and the band intensity was quantified with ImageJ software. For Co-IP experiments, the protein supernatant, lysed using Western and IP cell lysate (Beyotime, China) with 1% PMSF, underwent pre-incubation with Protein A+G agarose beads at 4°C for 30 minutes to minimize non-specific binding. Subsequently, the mixture was evenly distributed into four 1.5 mL tubes. Samples from three tubes were then subjected to overnight incubation with magnetic beads pre-bound with rabbit IgG (Beyotime), anti-S100A2 (Abcam), and anti-FoxO1 (Affinity) on a shaker at 4°C, while the remaining tube was left untreated. Following washes with TBS (Beyotime), the proteins were dissolved in loading buffer and subsequently heated at 95°C for 5 minutes for subsequent immunoblotting analysis.

### Transcriptome sequencing and bioinformatics analysis

After transfection, HK-2 cells were collected and suspended in 1 mL of Trizol reagent. The samples were then sent to Personalbio (Shanghai, China) for transcriptome sequencing. The resulting data were analyzed and visualized using the R software packages "clusterProfiler," "DOSE," "org.Hs.eg.db," "stringr," and "ggplot2."

### Immunofluorescence staining

HK-2 cells were first fixed onto slides, followed by washing and permeabilization with 0.1% Triton X100, and blocking with goat serum. Then cells were placed at 4°C and exposed to primary antibodies overnight, including anti-E-cadherin (Abcam), anti-type I collagen (Proteintech), anti-type III collagen (Proteintech), anti-N-cadherin (Abcam), S100A2 (Abcam), FoxO1 (Affinity), p-FoxO1 (Ser256, Affinity), anti-α-SMA (Santa Cruz), and anti-p-Smad2/3 (Thr8, Abcam). Subsequent incubation with secondary antibodies conjugated with Alexa Fluor 488 or 588 (Invitrogen, USA) followed. Finally, DAPI was used for nuclei visualization, and microscopy (Leica) was utilized for image capture.

### EMT detection

To evaluate epithelial-to-mesenchymal transition (EMT), HK2 cells were cultured under standard conditions using DMEM/DF12 medium and treated with TGF-β1 to induce EMT. Additionally, a mouse model of renal fibrosis was established. Morphological changes in the cells were assessed using optical microscopy. EMT markers such as E-cadherin, N-cadherin, vimentin, and α-SMA, were analyzed at both cellular and tissue levels using Western blotting with specific antibodies. Immunofluorescence staining was employed to examine the localization and expression of EMT markers, while immunohistochemistry (IHC) was utilized to determine the expression of these markers in tissue samples. RNA was extracted and reverse transcribed into cDNA, followed by quantitative PCR to quantify the mRNA levels of EMT-related markers. This comprehensive approach allowed for detailed assessment of EMT at various biological levels.

### Statistical analysis

Data were analyzed statistically using GraphPad Prism 9.0. The comparison between two groups was conducted using the t-test, while one-way analysis of variance (ANOVA) was employed for comparisons among multiple groups. Statistical significance was considered when *P* < 0.05.

## Results

### Enhanced S100A2 expression in three RIF mouse models

**Fig. 1:**
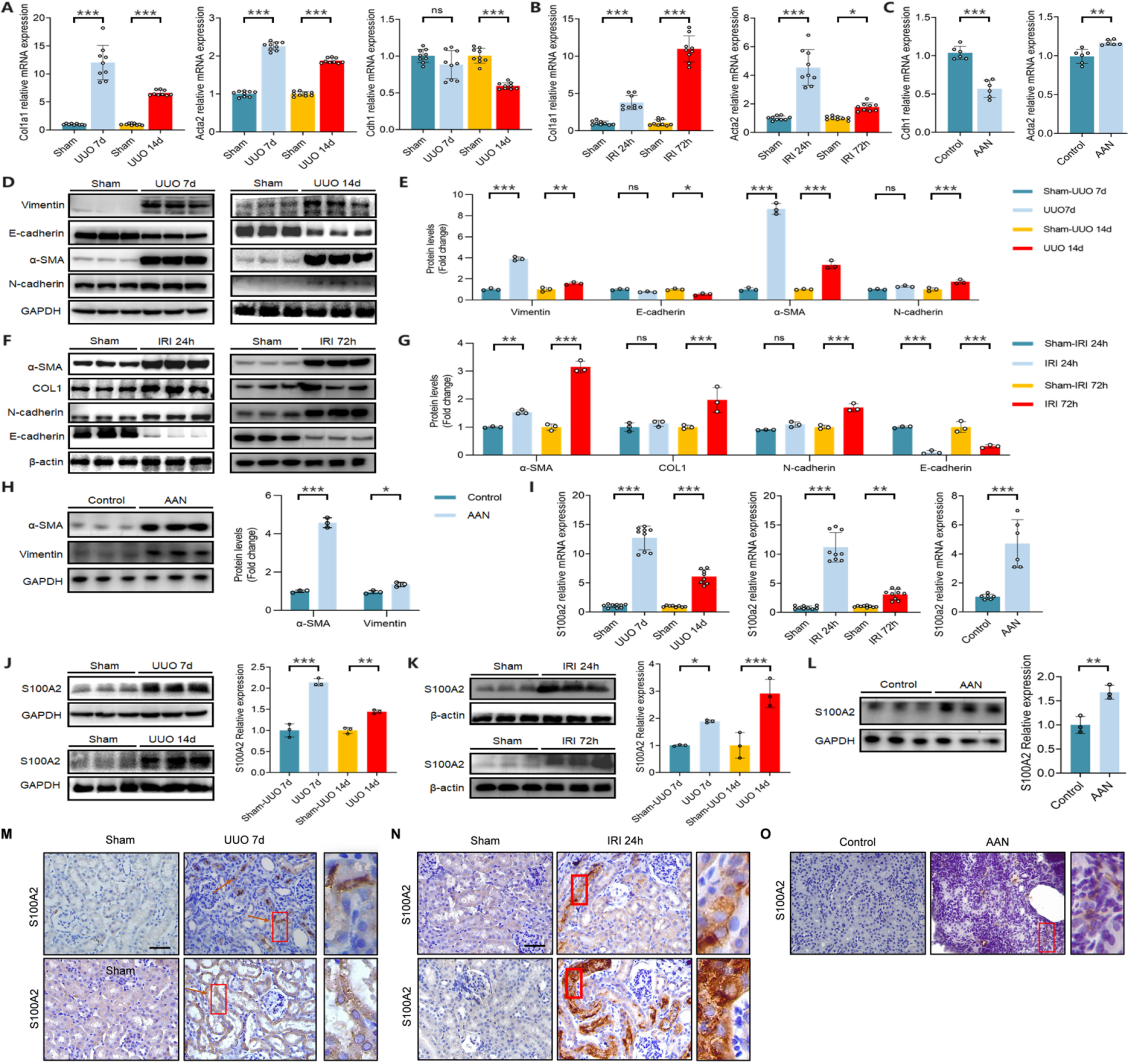
S100A2 is highly expressed in three RIF mouse models. RT-qPCR shows (**A**) upregulation of type I collagen and α-SMA mRNA and downregulation of E-cadherin mRNA in UUO-induced fibrosis; (**B**) high expression of type I collagen and α-SMA mRNA in IRI mice; (**C**) elevated levels of α-SMA and decreased E-cadherin mRNA levels in AAN mice. Immunoblotting analysis for fibrosis-related protein levels in (**D**-**E**) UUO, (**F**-**G**) IRI, and (H) AAN mice. (**I**) RT-qPCR analysis of S100A2 mRNA levels in mice after UUO, IRI, and AAN treatment. (**J**-**L**) Immunoblotting analysis for S100A2 protein levels in mice after UUO, IRI, and AAN treatment. (**M**-**O**) Representative micrographs showing S100A2 staining in UUO, IRI, and AAN mice. Scale bar, 50 μm. ^*^P < 0.05, ^**^P < 0.01, ^***^P < 0.001. ns, no statistical difference

UUO model is a validated classic model for RIF, wherein we explored the expression levels of S100A2. Renal IRI model is a classical, hypoxic-ischemic-induced RIF model. AAN is a progressive kidney disease caused by Chinese herbal medicine containing aristolochic acid, characterized by widespread cell apoptosis and interstitial fibrosis. We investigated the expression of S100A2 in these three RIF models. HE and Masson were utilized to assess tubulointerstitial injury and interstitial ECM accumulation in the kidney. As depicted in Supplementary Fig. 1A-B, demonstrated that, in comparison to the Sham group, discernible structural damage was observed in the kidney tissue on the 7th day post-UUO surgery, characterized by excessive dilation of renal tubules, extensive shedding of tubular brush borders, and notable shrinkage of glomeruli. By day 14 post-UUO, the renal tissue damage became even more pronounced. Compared to the sham group, the IRI group (24 and 72 hours post-IRI surgery) exhibited significant renal pathological damage (Supplementary Fig. 1D-E). Masson staining revealed a notable accumulation of total collagen in the kidneys of both UUO and IRI groups, manifested by an expansion in the area of blue-stained regions within the renal interstitium (Supplementary Fig. 1A-D). The AAN group was observed pronounced interstitial fibrosis compared to the solvent control group, accompanied by tubular injury, tubular dilation, basement membrane detachment, glomerular atrophy, and loss of tubular epithelial cells (Supplementary Fig. 1G). N-cadherin, α-SMA, Vimentin, and collagen are key factors contributing to renal fibrosis. Further research unveiled that UUO mice showcased an accumulation of type I collagen, upregulation of α-SMA, N-cadherin, and Vimentin, alongside downregulation of E-cadherin (Fig. 1A, D-E). In the IRI model, there was a downregulation of E-cadherin expression, accompanied by an upregulation of N-cadherin, type I collagen, and α-SMA expression (Fig. 1B, F-G). Similarly, AAN induced upregulation of α-SMA and Vimentin and downregulation of E-cadherin (Fig. 1C, H). In summary, we observed prominent fibrotic alterations in renal tissue across the UUO, IRI, and AAN models, concurrently accompanied by EMT.

Next, we further investigated expression and localization of S100A2 in renal tissues. Compared to the sham group or control group, both mRNA (Fig. 1I) and protein (Fig. 1J-L) expression of S100A2 in the kidneys of fibrotic mice were significantly upregulated. Immunohistochemistry (IHC) results also confirmed high expression of S100A2 in fibrotic kidneys, primarily localized in the cytoplasm of renal tubular epithelial cells (Fig. 1M-O). These findings suggested that during the EMT and RIF processes induced by UUO, IRI, and aristolochic acid, there was an increase in the expression of S100A2 in epithelial cells.

### High expression of S100A2 in TGF-β1-mediated *in vitro* fibrosis model

**Fig. 2:**
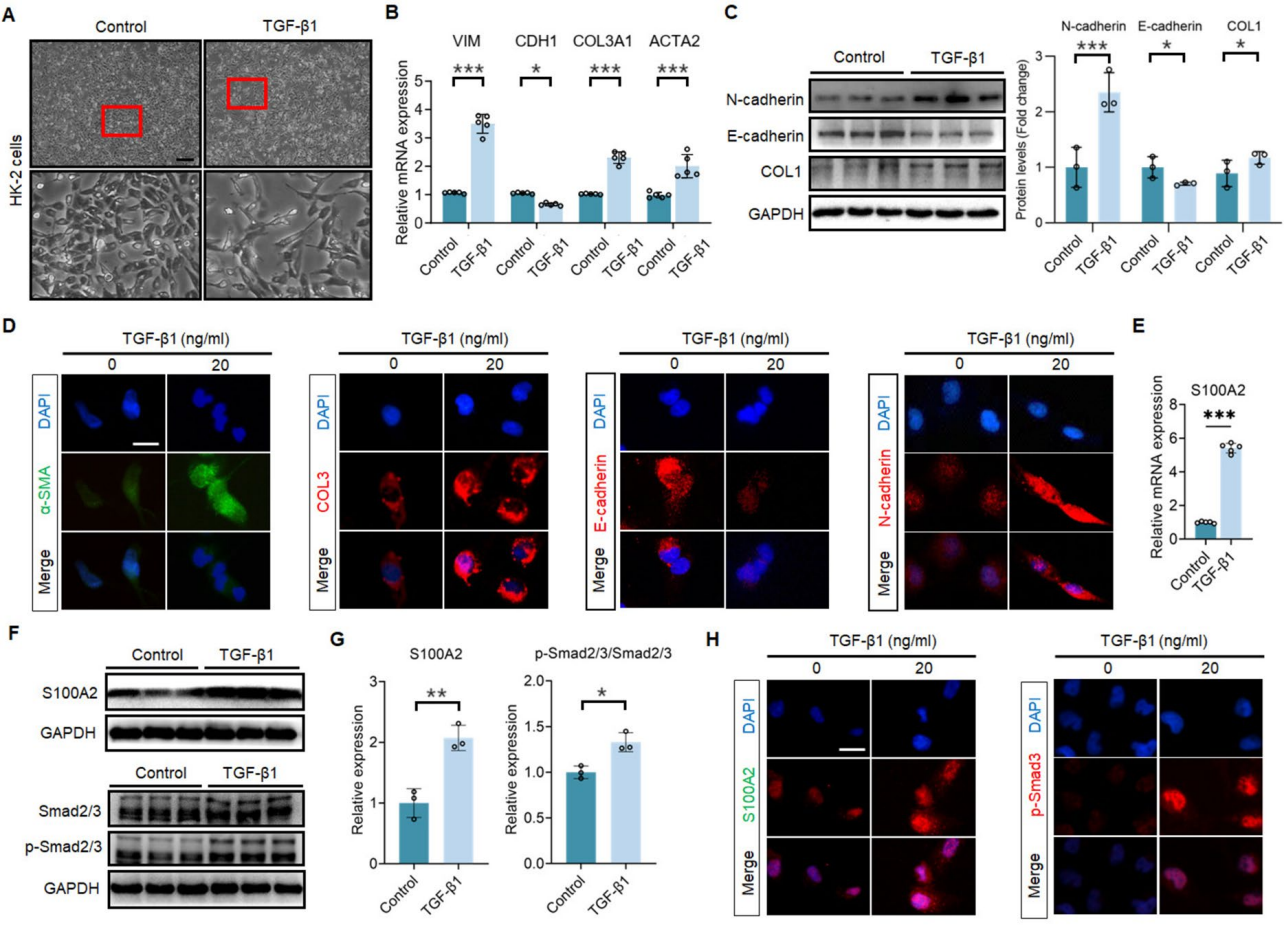
S100A2 is highly expressed in TGF-β1-mediated HK-2 cells. (**A**) Representative micrographs showing morphological changes of HK-2 cells before and after TGF-β1 treatment. Scale bar, 200 μm. (**B**) RT-qPCR analysis of EMT and ECM gene expression in TGF-β1-stimulated HK-2 cells. (**C**) Induction of EMT and ECM proteins in TGF-β1-stimulated HK-2 cells. (**D**) IF staining showing localization of EMT and ECM markers in TGF-β1-stimulated HK-2 cells. Scale bar, 25 μm. (**E**) RT-qPCR analysis of S100A2 mRNA levels in TGF-β1-stimulated HK-2 cells. Representative immunoblotting (**F**) and quantification data (**G**) showing induction of S100A2 and p-Smad2/3 proteins in TGF-β1-stimulated HK-2 cells. (**H**) IF showing localization of S100A2 and p-Smad2/3 in TGF-β1-stimulated HK-2 cells. Scale bar, 25 μm. ^*^P < 0.05, ^**^P < 0.01, ^***^P < 0.001

To investigate whether S100A2 is involved in fibrosis process, HK-2 cells were treated with 20 ng/ml recombinant human TGF-β1 for 48 hours. The results showed that TGF-β1 significantly promoted morphological changes in HK-2 cells, transitioning from epithelial cell morphology (cuboidal or flat) to mesenchymal cell morphology (spindle-shaped), with thinner cytoplasm, increased cell volume, and elongated cell protrusions (Fig. 2A). Further analysis manifested that TGF-β1 treatment augmented mRNA expression of Vimentin, type III collagen, and α-SMA, while decreased E-cadherin mRNA expression (Fig. 2B). Consistently, other experimental results also demonstrated upregulation of N-cadherin and type I collagen expression and downregulation of E-cadherin expression following TGF-β1 treatment (Fig. 2C). IF staining revealed that TGF-β1 recombinant protein enhanced cytoplasmic levels of α-SMA, type III collagen, and N-cadherin, while inhibiting cytoplasmic expression of E-cadherin in HK-2 cells (Fig. 2D). These results indicated that TGF-β1 induced significant EMT in HK-2 cells. Further investigation elucidated that TGF-β1 treatment activated phosphorylation of Smad2/3 signaling, promoting mRNA and protein expression of S100A2 (Fig. 2E-G). However, the inhibition of TGF-β1 effectively blocked the expression of S100A2, as demonstrated in Supplementary Fig. 2A. Furthermore, exposure to hypoxic conditions induced by treatment with CoCl2, resulted in a significant increase in S100A2 expression in HK2 cells, as shown in Supplementary Fig. 2B. Immunofluorescence results showed elevated cytoplasmic and nuclear expression of S100A2 in the treatment group, with phosphorylated Smad2/3 translocating from the cytoplasm to the nucleus (Fig. 2H).

### Increased S100A2 in various fibrotic kidney diseases

**Fig. 3:**
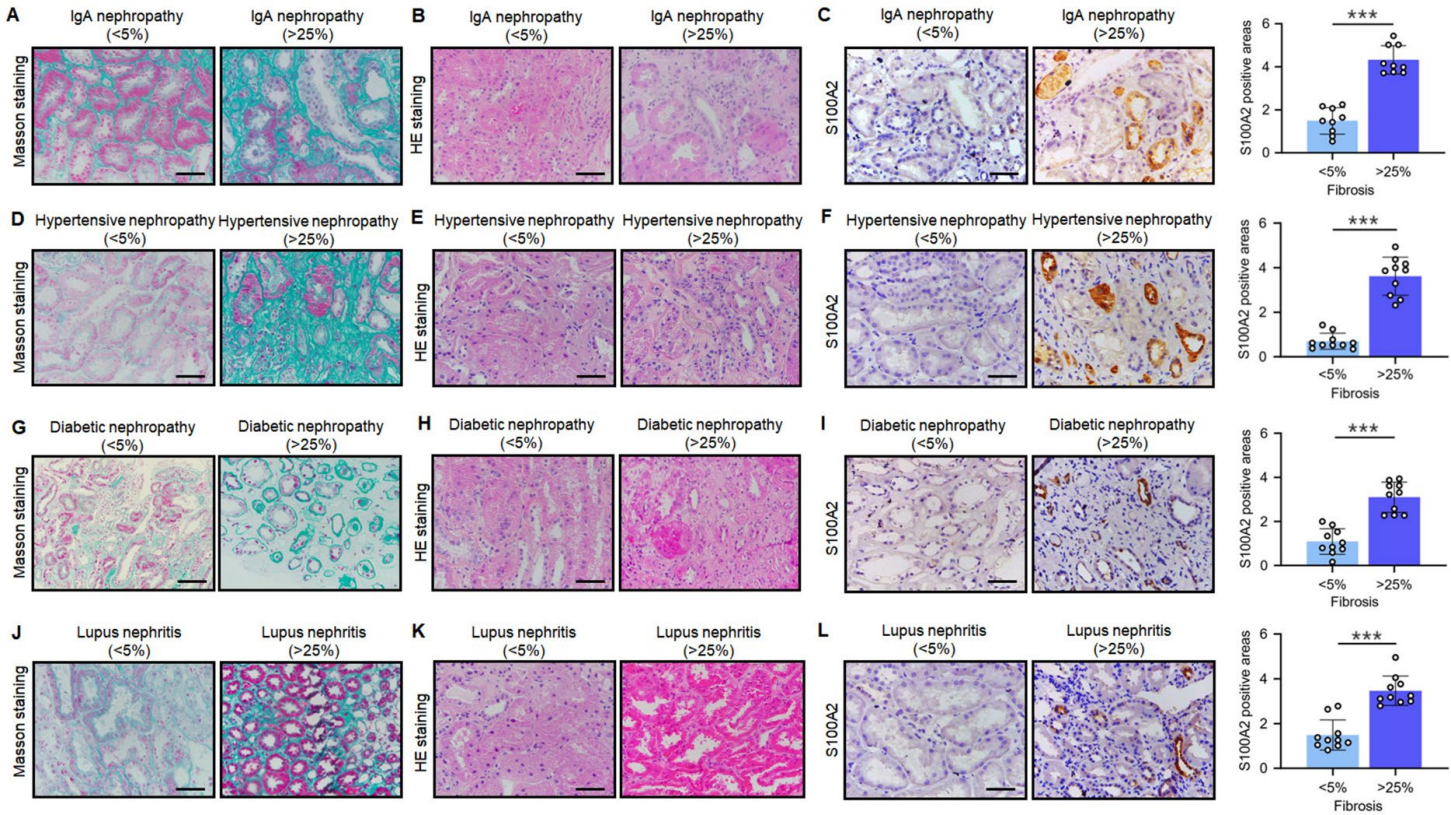
Compared to the low fibrosis group, S100A2 expression increases in different kidney diseases with high fibrosis. In IgA nephropathy, analysis of (**A**) renal injury (**B**) fibrosis and (**C**) S100A2 levels in low and high fibrosis groups. In hypertensive nephropathy, analysis of (**D**) renal injury (**E**) fibrosis and (**F**) S100A2 levels in low and high fibrosis groups. In diabetic nephropathy, analysis of (**H**) renal injury (**I**) fibrosis and (**J**) S100A2 levels in low and high fibrosis groups. In lupus nephropathy, analysis of (**K**) renal injury (**L**) fibrosis and (**M**) S100A2 levels in low and high fibrosis groups. Scale bar, 50 μm. ^***^P < 0.001

To further validate the expression of S100A2 in fibrotic diseases, samples were collected from four common kidney diseases: IgA nephropathy, hypertensive nephropathy, diabetic nephropathy, and lupus nephropathy. These samples were divided into low fibrosis (<5%) and high fibrosis (>25%) groups. Masson staining results, as depicted in Fig. 3A, D, G, J, revealed a significant increase in collagen accumulation in the high fibrosis groups of all four kidney diseases, indicating severe fibrotic lesions. HE staining demonstrated severe renal damage in the high fibrosis groups, characterized by tubular lumen dilation, interstitial inflammatory cell infiltration, among other pathological changes (Fig. 3B, E, H, K). Importantly, compared to the low fibrosis groups, the high fibrosis groups of several kidney diseases exhibited significantly elevated expression of S100A2 in kidney tissues (Fig. 3C, F, I, L), suggesting the potential involvement of S100A2 in fibrosis.

### S100A2 knockdown inhibits TGF-β1-mediated renal tubular epithelial cell EMT and ECM deposition

**Fig. 4:**
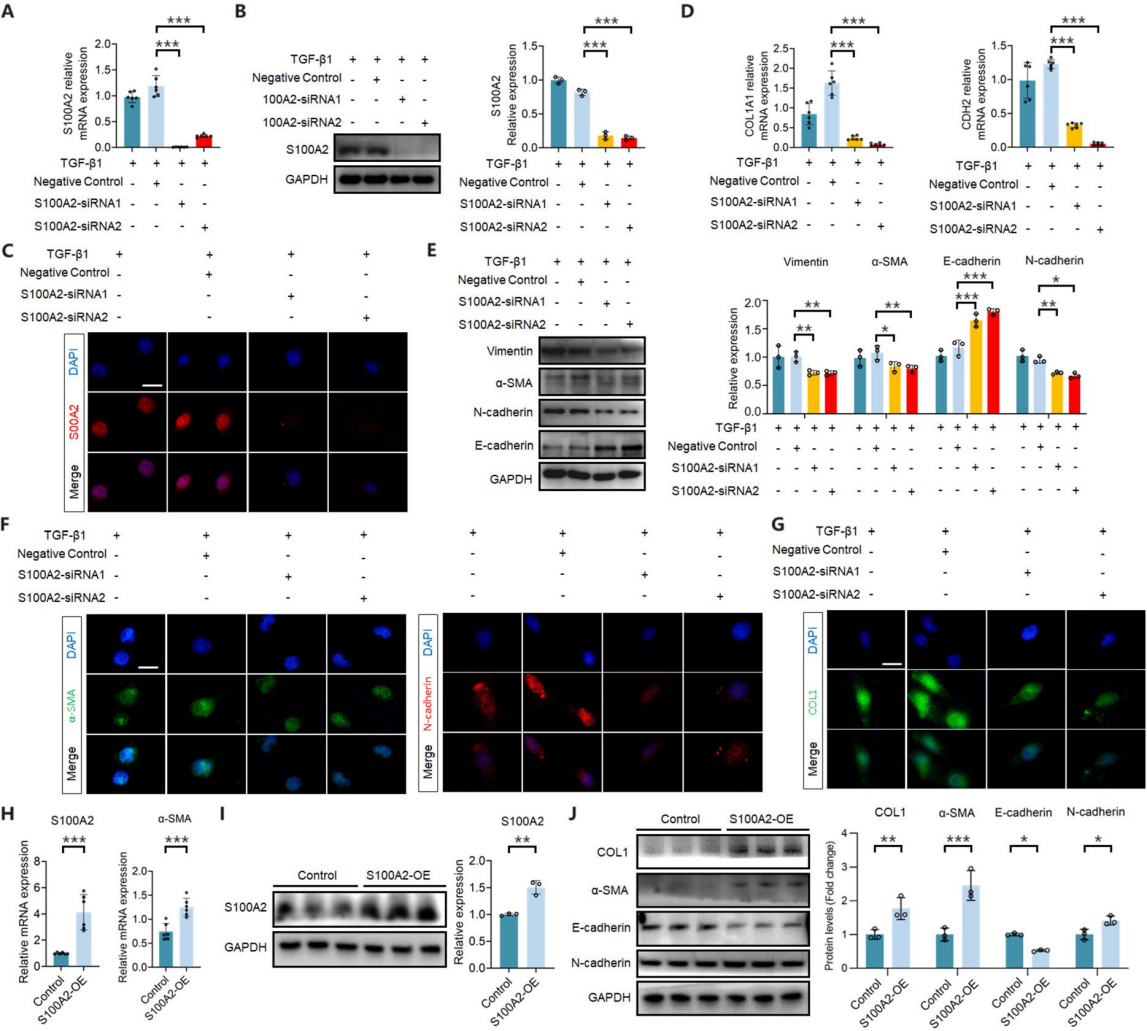
S100A2 knockdown inhibits TGF-β1-induced EMT and ECM in HK-2 cells, while S100A2 overexpression promotes EMT and ECM in HK-2 cells. S100A2-siRNA successfully inhibits S100A2 (**A**) mRNA and (**B**-**C**) protein levels. Scale bar, 25 μm. (**D**) S100A2 knockdown blocks type I collagen and N-cadherin mRNA expression in TGF-β1-stimulated HK-2 cells. (**E**) S100A2 knockdown inhibits EMT and ECM markers’ protein expression in TGF-β1-stimulated HK-2 cells. S100A2 knockdown decreases (**F**-**G**) cytoplasmic expression of α-SMA, N-cadherin, and type I collagen induced by TGF-1 stimulation in HK-2 cells. (**H**-**I**) S100A2 overexpression enhances α-SMA mRNA expression in HK-2 cells. (**J**) S100A2 overexpression induces expression of type I collagen, α-SMA, N-cadherin, and suppresses E-cadherin protein expression in HK-2 cells. ^*^P < 0.05, ^**^P < 0.01, ^***^P < 0.001

The process of renal tubular epithelial cell EMT plays a crucial role in the development and progression of RIF. To investigate the role of S100A2 in TGF-β1-mediated renal tubular epithelial cell EMT, we applied small interfering RNA to target two different sites of S100A2 to block its expression. The results revealed successful inhibition of S100A2 mRNA and protein expression (Fig. 4A-B). IF staining confirmed a decrease in S100A2 expression intensity (Fig. 4C). α-SMA is a marker of myofibroblast cell activation and serves as a cytoskeletal protein. Vimentin is the major intermediate filament protein, playing an important role in connecting the cell membrane with the nucleus. E-cadherin and N-cadherin are a class of important adhesion molecules located on the cell membrane, regulating cell adhesion, and maintaining the integrity of epithelial cell structure and function. Decreased E-cadherin and increased N-cadherin interrupt the connection between epithelial cells, allowing cells to migrate, acquire mesenchymal-like changes, and undergo EMT. Inhibiting S100A2 expression reduced the gene expression of type I collagen and adhesion protein N-cadherin (Fig. 4D), decreased the expression of cytoskeletal protein Vimentin and α-SMA and adhesion protein N-cadherin, and promoted the levels of epithelial adhesion protein E-cadherin (Fig. 4E). IF analysis also demonstrated similar results (Fig. 4F-G). In conclusion, knocking down S100A2 inhibited the EMT process and ECM deposition induced by TGF-β1.

### S100A2 overexpression promotes TGF-β1-mediated EMT and ECM deposition in HK2 cells

Even though our results suggest that inhibiting S100A2 has antagonistic effects on EMT and RIF, further confirmation is needed by overexpressing S100A2 to determine whether it exacerbates EMT and RIF. In this study, recombinant human S100A2 protein was introduced into HK-2 cells to activate S100A2 (Fig. 4H-I). We observed that increased expression of S100A2 enhanced the transcription levels of activated myofibroblast marker α-SMA (Fig. 4H). Moreover, upregulation of S100A2 expression not only elevated α-SMA protein expression but also promoted the expression of extracellular matrix component type I collagen while reducing the expression of intercellular adhesion protein E-cadherin and enhancing N-cadherin expression (Fig. 4J).

### FoxO1 is a potential target of S100A2

**Fig. 5:**
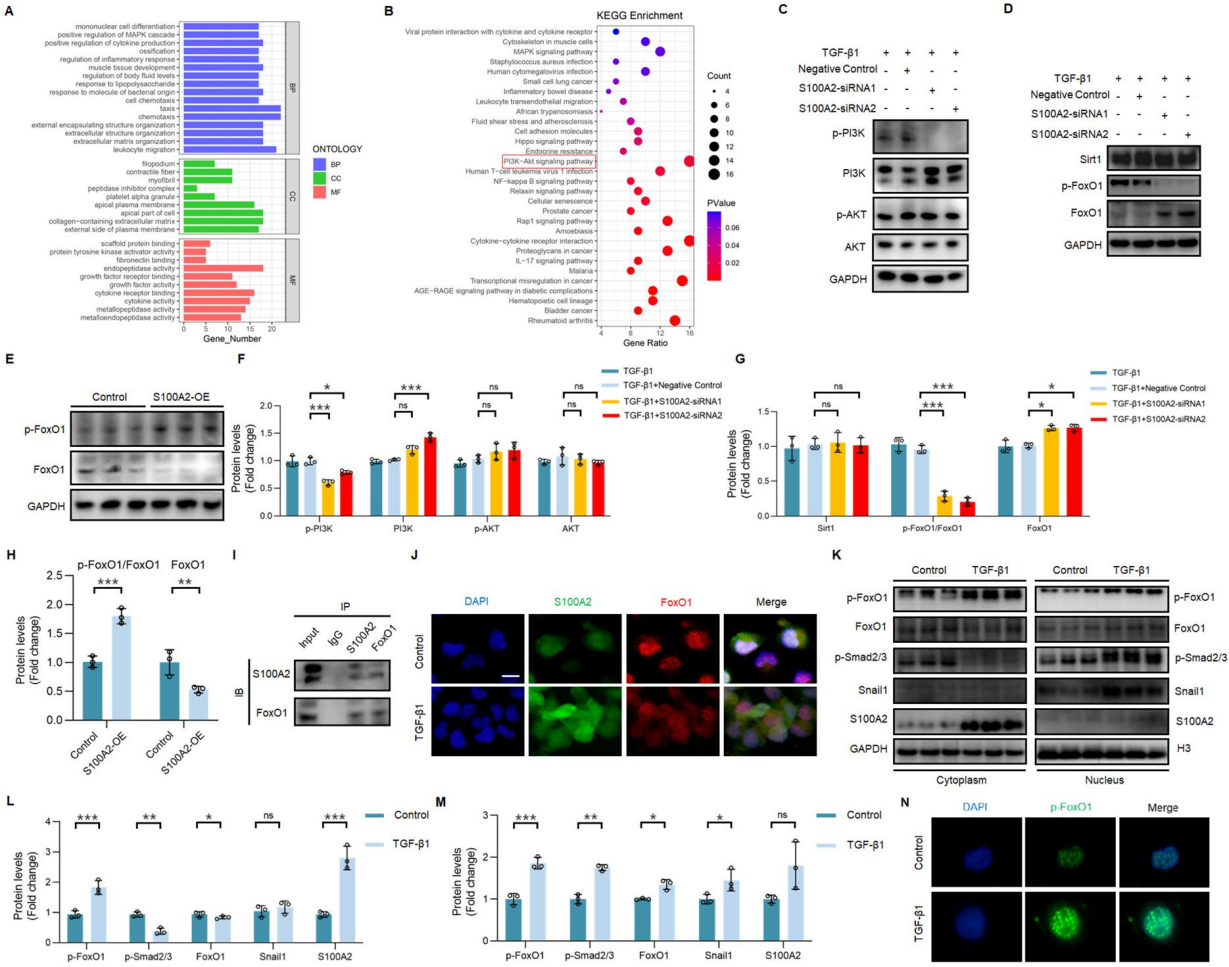
FoxO1 is a potential target of S100A2. (**A**) GO and (**B**) KEGG enrichment analyses for differentially expressed genes in TGF-β1-stimulated HK-2 cells with S100A2 knockdown. BP, CC, MF denote biological processes, cellular components, and molecular functions, respectively. S100A2 knockdown blocks (**C**, **F**) PI3K and activates (**D**, **G**) FoxO1. (**E**, **H**) Overexpression of S100A2 inhibits FoxO1 levels. (**I**) Co-IP demonstrates the interaction between S100A2 and FoxO1. (**J**) IF confirms the co-localization of S100A2 and FoxO1. Scale bar, 25 μm. (**K**-**M**) Immunoblot analysis of nuclear and cytoplasmic levels of p-FoxO1, FoxO1, p-Smad2/3, Snail1, and S100A2 in TGF-β1-stimulated HK-2 cells. (**N**) The effect of TGF-β1 treatment on the fluorescence intensity of p-FoxO1 in HK2 cells. ^*^P < 0.05, ^**^P < 0.01, ^***^P < 0.001. ns, no statistical difference

Our research results have shown that S100A2 regulate the EMT process and renal RIF, but the downstream targets and specific mechanisms of S100A2 are not clear. Therefore, we performed transcriptome sequencing. Sequencing analyses revealed that following the inhibition of S100A2, GO enrichment analyses indicated that the differentially expressed genes were predominantly enriched in cellular components, biological processes, and molecular functions related to leukocyte migration, chemotaxis, the apical part of the cell, the collagen-containing extracellular matrix, and endopeptidase activity (Fig. 5A), suggesting that S100A2 may be involved in regulating matrix synthesis and remodeling, tissue growth, development, and migration. In addition, KEGG enrichment analysis (Fig. 5B) further analyzed related biological pathways, including immune and metabolic diseases, inflammation-related signaling (IL-17, AGE-RAGE, NF-κB), and proliferation and migration-related signaling pathways (PI3K-AKT, cell adhesion molecules).

Our previous studies have discovered that S100A2 regulates EMT and promotes collagen deposition, consistent with the cascading effects on proliferation and migration-related signaling pathways indicated by the sequencing results. Therefore, we further investigated the effect of S100A2 knockdown on the PI3K/AKT pathway. Interestingly, although S100A2 knockdown decreased phosphorylated PI3K, it did not activate AKT (Fig. 5C, F). It has been demonstrated that FoxO1 is an essential signaling component in the PI3K pathway, and its phosphorylation induced FoxO1 inactivation, while deacetylation by the enzyme Sirt1 enhances FoxO1 activity (Chen et al. 2023a). Recent literature reported that targeting PI3K/FoxO1 alleviated liver fibrosis (Yan et al. 2021). Considering that FoxO1, as a key transcription factor, may be essential for the S100A2-mediated EMT process, we examined its expression and activity. The results showed that FoxO1 was inhibited in three RIF models, and *in vitro* treatment with TGF-β1 decreased FoxO1 (Supplementary Fig. 3A-E). It was found that S100A2 knockdown weakened the protein expression of phosphorylated FoxO1, thereby activating FoxO1 (Fig. 5D, G, Supplementary Fig. 3F, H). Conversely, overexpression of S100A2 promoted FoxO1 phosphorylation, thereby inhibiting its activity (Fig. 5E, H, Supplementary Fig. 3G, I). These results indicated that FoxO1 may be a potential regulatory target of S100A2.

### S100A2 antagonizes FoxO1, driving EMT and ECM deposition

To further validate the interaction between S100A2 and FoxO1, we conducted Co-IP experiments. As shown in Fig. 5I, S100A2 interacts with FoxO1, suggesting their collaborative involvement in similar biological functions. IF staining further clarified their subcellular localization. TGF-β1 stimulation in HK2 cells suppressed the nuclear expression of FoxO1 while promoting cytoplasmic expression of S100A2. Additionally, partial co-localization between S100A2 and FoxO1 was observed, indicating potential protein-protein interaction or functional relevance (Fig. 5J). Furthermore, the nuclear and cytoplasmic expression of S100A2 and FoxO1 during TGF-β1-induced fibrosis progression were determined. The results revealed that TGF-β1 treatment induced the translocation of its downstream key transcription factors p-Smad2/3 and Snail1 from the cytoplasm to the nucleus, enhancing cytoplasmic localization of S100A2 and nuclear expression of FoxO1 (Fig. 5K-**N**).

**Fig. 6:**
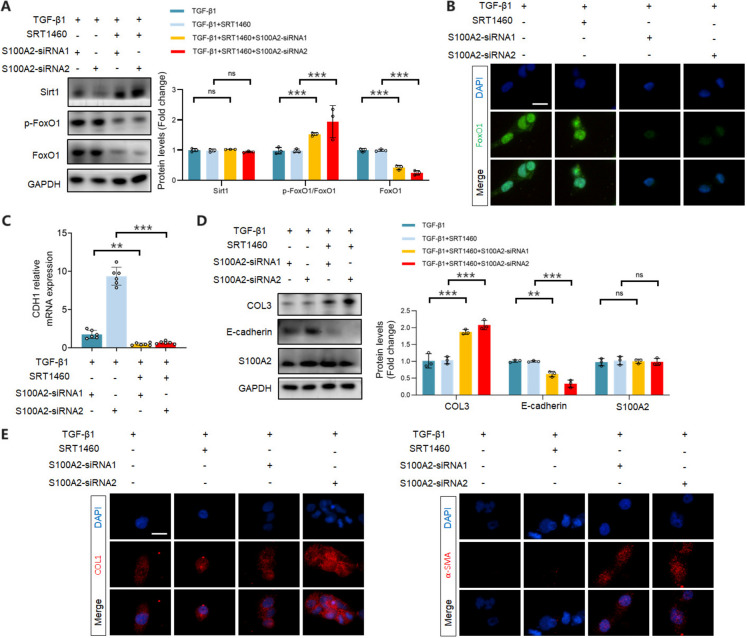
Inhibition of FoxO1 reverses the suppressive effects of S100A2 knockdown on EMT and ECM. Sirt1 agonist SRT1460 inhibits (**A**) FoxO1 protein expression and (**B**) nuclear localization. Scale bar, 25 μm. (**C**) Inhibition of FoxO1 abolishes the upregulation of E-cadherin in TGF-β1-stimulated HK-2 cells following S100A2 knockdown. (**D**) Inhibition of FoxO1 reverses the upregulation of E-cadherin and downregulation of type III collagen in TGF-β1-stimulated HK-2 cells following S100A2 knockdown. (**E**) Inhibition of FoxO1 restores cytoplasmic localization of α-SMA and type I collagen. Scale bar, 25 μm. ^**^P < 0.01, ^***^P < 0.001. ns, no statistical difference

Then, SRT1460, an effective Sirt1 agonist was employed to inhibit FoxO1 expression in rescue experiments (Fig. 6A). The results demonstrated that SRT1460 effectively counteracted the S100A2 knockdown-induced nuclear expression of FoxO1 under TGF-β1 treatment (Fig. 6B). Besides, SRT1460 abolished the elevated E-cadherin mRNA expression induced by S100A2 knockdown (Fig. 6C), and restored the Type III collagen and reduced E-cadherin (Fig. 6D). IF experiments further revealed that pharmacological inhibition of FoxO1 by SRT1460 eliminated the cytoplasmic expression inhibition of type I collagen and α-SMA, interstitial markers induced by S100A2 knockdown (Fig. 6E). In summary, pharmacological inhibition of FoxO1 by SRT1460 alleviated the inhibitory effect of S100A2 knockdown on EMT and ECM.

## Discussion

Renal fibrosis manifests as the aberrant proliferation and deposition of fibrous tissue in response to kidney injury, culminating in glomerulosclerosis and the formation of renal fibrosis (Yan et al. 2021). Its etiology is multifactorial, encompassing factors such as trauma, infection, inflammation, vascular disorders, and immune reactions (Zhang and Zhang 2020). These inciting factors inflict damage on tubular epithelial cells, prompting activation of fibroblasts and macrophages, and instigating inflammatory and fibrotic cascades (Fu et al. 2022; Li et al. 2019; Shen and Lv 2022). Initially, fibrosis may serve reparative purposes, yet sustained injury and stimulation can exacerbate fibrotic activity, leading to extensive tissue fibrosis and compromise of renal structure and function (Yamashita and Kramann 2024). CKD mediated by renal fibrosis is another threat to human health following cardiovascular diseases, tumors, and diabetes. It has been reported that the incidence of CKD in China is approximately 10.8%, with a steady upward trend. CKD patients confront the prospect of lifelong dialysis or kidney transplantation, imposing significant socioeconomic ramifications. Currently, anti-inflammatory, antioxidant stress, and anti-fibrotic therapies are the main strategies for renal fibrosis (Si et al. 2021). However, efficacious and safe anti-renal fibrosis interventions remain elusive. Therefore, comprehensive elucidation of the molecular underpinnings driving renal fibrosis progression, identification of potential therapeutic targets, and advancement of diagnostic, treatment, and prognostic modalities are imperative pursuits in combating fibrotic disorders.

S100 calcium-binding protein A2 has long been recognized for its role in tumors (Chen et al. 2023b; Li et al. 2020; Masuda et al. 2016; Yan et al. 2022; Zhang et al. 2022), but its relationship with renal fibrosis and its mechanism of action remain unknown. Our study filled this gap. Our research indicated that compared to sham surgery and solvent control groups, the expression of S100A2 in renal tissues is significantly increased in three classic CKD models, especially in the cytoplasm of severely damaged tubular epithelial cells. A recent study aligns intriguingly with our findings, demonstrating elevated expression of S100A2 in patients with pulmonary fibrosis. Moreover, the study revealed that knockdown of S100A2 promotes EMT in lung epithelial cells by inhibiting downregulated kinases, namely p-GSK-3β and β-Catenin, and by antagonizing the Wnt/β-Catenin signaling pathway (Huang et al. 2021). Originally, the scientific community generally embraced the concept of complete EMT in renal tissue. However, as evidence supporting partial EMT's role in promoting fibrosis has accumulated, it has garnered widespread acceptance among researchers. Specifically, PTECs undergoing phenotypic transformation do not transform into interstitial fibroblasts, but remain in the tubules, displaying partial EMT status, exhibiting both epithelial and mesenchymal characteristics (Lovisa et al. 2016). We investigated whether S100A2 is associated with renal fibrosis by conducting experiments on HK-2 renal tubular epithelial cell line, which is most sensitive to injury stimuli. In our *in vitro* experiments, we utilized recombinant protein TGF-β1 to stimulate HK-2 cells, inducing a fibrosis-like phenotype. The results showed that TGF-β1 mediated morphological changes in HK-2 cells, with cells transitioning from tightly arranged cobblestone-like to scattered spindle-shaped, with increased expression of type I collagen and type III collagen, activated myofibroblast marker α-SMA, increased levels of interstitial markers Vimentin and N-cadherin, along with reduced expression of the epithelial marker E-cadherin, indicating the occurrence of EMT and ECM. Additionally, there was a notable increase in the expression of S100A2 in the renal cortex of patients with RIF. Our study elucidated a likely correlation between S100A2 and RIF, underscoring its significance in the pathological process.

To delve into the specific role of S100A2 in renal fibrosis, we conducted *in vitro* experiments involving both gene knockdown and overexpression of S100A2. Inhibiting S100A2 with siRNA reduced the expression of type I collagen and adhesion proteins N-cadherin and the levels of cytoskeletal protein Vimentin and α-SMA, boosted the levels of the epithelial protein E-cadherin induced by TGF-β1, while overexpression of S100A2 had the opposite effect. These findings validated the pro-EMT role of S100A2 from both perspectives. Previous studies have suggested that antagonizing EMT is an effective anti-fibrotic approach (Lovisa et al. 2016), and S100A2 holds promise as a potential therapeutic target for RIF and other fibrosis-related diseases due to its pro-EMT properties. Sequencing analysis identified PI3K/AKT as potential signaling pathway. Recent research by Li et al. revealed that inhibiting PI3K/AKT activity with the compound herbal medicine SHT successfully suppressed renal fibrosis induced by 5/6 nephrectomy (Li et al. 2023). Additionally, numerous studies have found that PI3K/AKT accelerated fibrosis progression by regulating cell proliferation, EMT, apoptosis, oxidative stress, and inflammation (Feng et al. 2022; Si et al. 2021; Yang et al. 2023). Importantly, we observed that S100A2 knockdown led to the deactivation of the PI3K/FoxO pathway, which has recently been found to be associated with EMT in peritoneum. Co-IP and IF revealed the interaction and co-localization of S100A2 with FoxO1, suggesting their potential collaboration in biological functions.

Several studies have underscored FoxO1's critical involvement in fibrosis and its close connection with fibrosis-related signaling pathways. For example, adenoviral overexpression of FoxO1 in wild-type mice promoted liver fibrosis and hepatic stellate cell activation induced by CCL4 injection, whereas this effect was blocked in mice lacking liver-specific TGF-β1 (Pan et al. 2024). Tissue-specific overexpression of FoxO1 in transgenic mouse models conferred renal protection, partly by attenuating diabetes-induced increases in Thioredoxin Interacting Protein (TXNIP) and reducing Thioredoxin (TRX) levels, thereby partially reversing renal interstitial fibrosis and apoptosis (Ji et al. 2019). As highlighted in a comprehensive review, FoxO1/3 play crucial inhibitory roles in stimulating fibroblast activation and the resulting synthesis of extracellular matrix, improving fibrosis levels in various organs including the heart, liver, lungs, and kidneys (Xin et al. 2018). In our study, we observed that the Sirt1 agonist SRT1460-mediated inhibition of FoxO1 impaired the blocking effects of S100A2 knockdown on EMT, consistent with the role of FoxO1 in fibrosis reported in previous literature. Our findings further supported the significance of FoxO1 in regulating the process of RIF.

While our study offers important insights into the function of S100A2 *in vitro*, it is important to acknowledge several limitations that may impact the generalizability of our findings. First, due to resource constraints, our analyses were conducted in a controlled *in vitro* environment, which may not fully replicate the complexity of in vivo biological systems. As a result, the interactions and regulatory mechanisms involving S100A2 in a whole organism context remain to be explored. Future studies incorporating *in vivo* approaches will be essential to validate our findings and elucidate the biological significance of S100A2 modulation in a more complex physiological framework.

In conclusion, our findings suggest that S100A2 antagonizes FoxO1 to promote EMT and ECM remodeling in renal PTECs, thereby partially elucidating the cellular and molecular mechanisms underlying RIF, which offers innovative strategies for both its prevention and treatment and establishes a theoretical framework for drug development and therapeutic interventions in conditions associated with EMT and fibrosis.

## Supplementary information

ESM 1 Supplementary Fig. 1 Assessment of renal injury and fibrosis in UUO and IRI mice, as well as in mice induced by aristolochic acid. (A-B) HE and Masson staining showing renal injury and total collagen accumulation in UUO mice. (C) IHC staining demonstrating the expression and localization of fibrosis-related markers (E-cadherin, α-SMA, Vimentin, type I collagen). (D-E) HE and Masson staining illustrating renal injury and total collagen accumulation in IRI mice. (F) IHC staining showing the expression and localization of fibrosis-related markers (α-SMA, type I collagen, E-cadherin, Vimentin). Assessment of renal injury and fibrosis in AAN mice. (G, H) HE and Masson staining revealing renal injury and total collagen accumulation in AAN mice. (I) IHC staining showing the expression and localization of fibrosis-related markers (α-SMA, type I collagen, E-cadherin, Vimentin). Scale bar, 50 μm. ^***^P < 0.001. (PNG 3.30 mb)

High resolution image (TIF 42.6 mb)

ESM 2 Supplementary Fig. 2 Expression of S100A2 in HK2 cells treated with TGF-β1 inhibitor or CoCl2. (A) The TGF-β1 inhibitor antagonized the protein expression of S100A2. (B) CoCl2 induced high expression of S100A2. (PNG 116 kb)

High resolution image (TIF 186 kb)

ESM 3 Supplementary Fig. 3 Protein and subcellular localization of FoxO1 under different treatments. FoxO1 inhibition is induced by (A) UUO, (B) IRI, and (C) aristolochic acid. (D) Inhibition of FoxO1 protein expression and (E) nuclear localization in TGF-β1- stimulated HK-2 cells. Scale bar, 25 μm. (F) S100A2 knockdown enhances nuclear localization of FoxO1, while (G) S100A2 overexpression disrupts its nuclear expression. Scale bar, 25 μm. (H) S100A2 knockdown inhibits nuclear localization of p-FoxO1, while (I) S100A2 overexpression promotes its nuclear expression. Scale bar, 25 μm. (PNG 401 kb)

High resolution image (TIF 941 kb)

ESM 4 (DOCX 14 kb)

## Data Availability

No datasets were generated or analysed during the current study.

## Abbreviations

AAN: aristolochic acid nephropathy
CKD: chronic kidney disease
Co-IP: co-immunoprecipitation
DAPI: 4', 6-diamidino-2-phenylindole
ECM: extracellular matrix
EMT: epithelial-to-mesenchymal transition
HE: hematoxylin and eosin
HK-2: Human kidney proximal tubular cells
IHC: immunohistochemistry
UUO: unilateral ureteral obstruction
IRI: ischemia-reperfusion injury
RT-qPCR: reverse transcription quantitative polymerase chain reaction
TGF-β1: transforming growth factor-β1
