# Supplementary figures and images for: S100A2 activation promotes interstitial fibrosis in kidneys by FoxO1-mediated epithelial-mesenchymal transition
Source: Cell Biol Toxicol. 2024 Oct 9;40(1):86. doi: 10.1007/s10565-024-09929-7 (PMC11464619; doi:10.1007/s10565-024-09929-7)

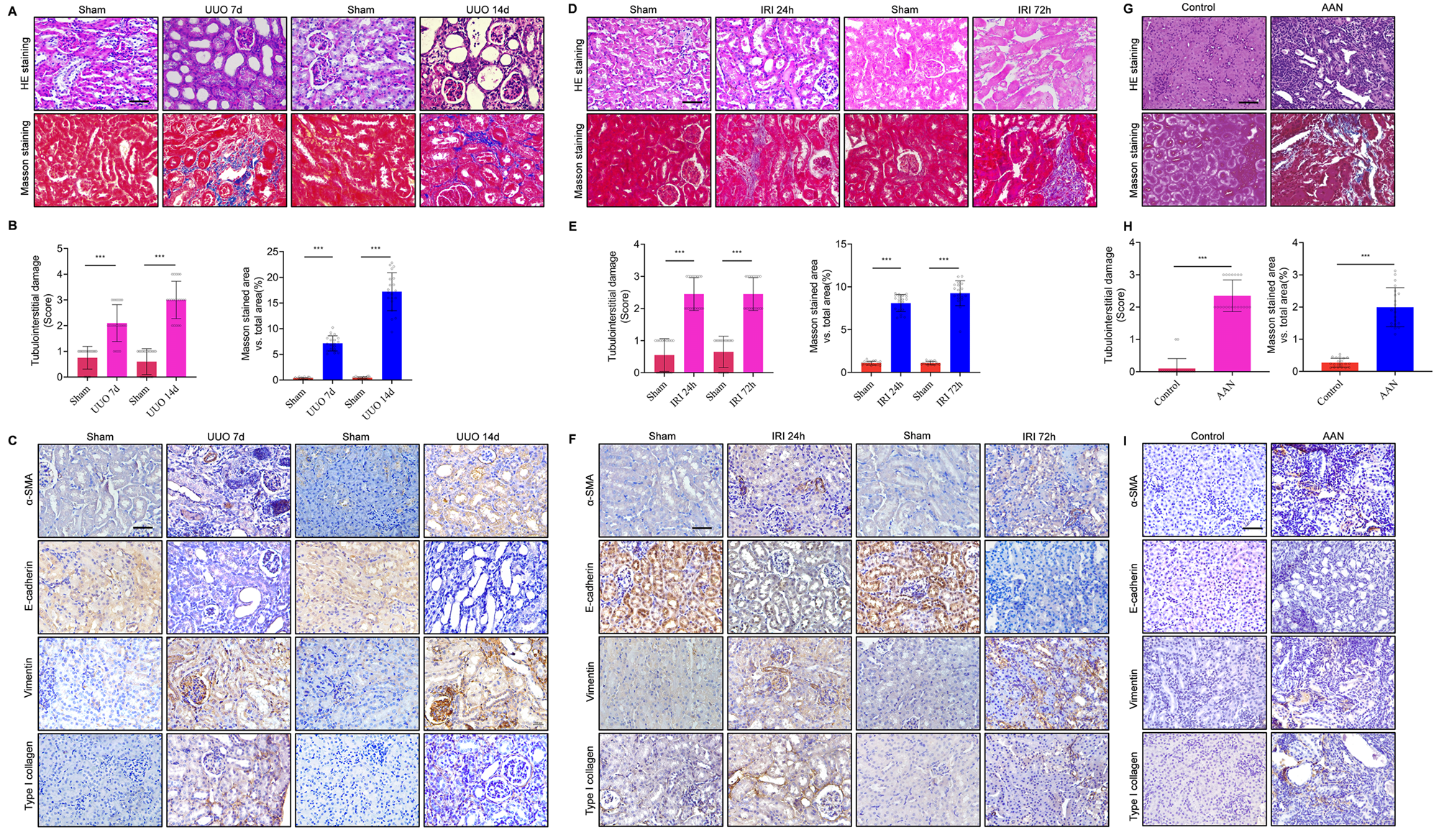

Supplement: Supplementary file 1 — Supplementary Fig. 1 Assessment of renal injury and fibrosis in UUO and IRI mice, as well as in mice induced by aristolochic acid. (A-B) HE and Masson staining showing renal injury and total collagen accumulation in UUO mice. (C) IHC staining demonstrating the expression and localization of fibrosis-related markers (E-cadherin, α-SMA, Vimentin, type I collagen). (D-E) HE and Masson staining illustrating renal injury and total collagen accumulation in IRI mice. (F) IHC staining showing the expression and localization of fibrosis-related markers (α-SMA, type I collagen, E-cadherin, Vimentin). Assessment of renal injury and fibrosis in AAN mice. (G, H) HE and Masson staining revealing renal injury and total collagen accumulation in AAN mice. (I) IHC staining showing the expression and localization of fibrosis-related markers (α-SMA, type I collagen, E-cadherin, Vimentin). Scale bar, 50 μm. ***P < 0.001. (PNG 3.30 mb) [file 10565_2024_9929_Fig7_ESM.png]

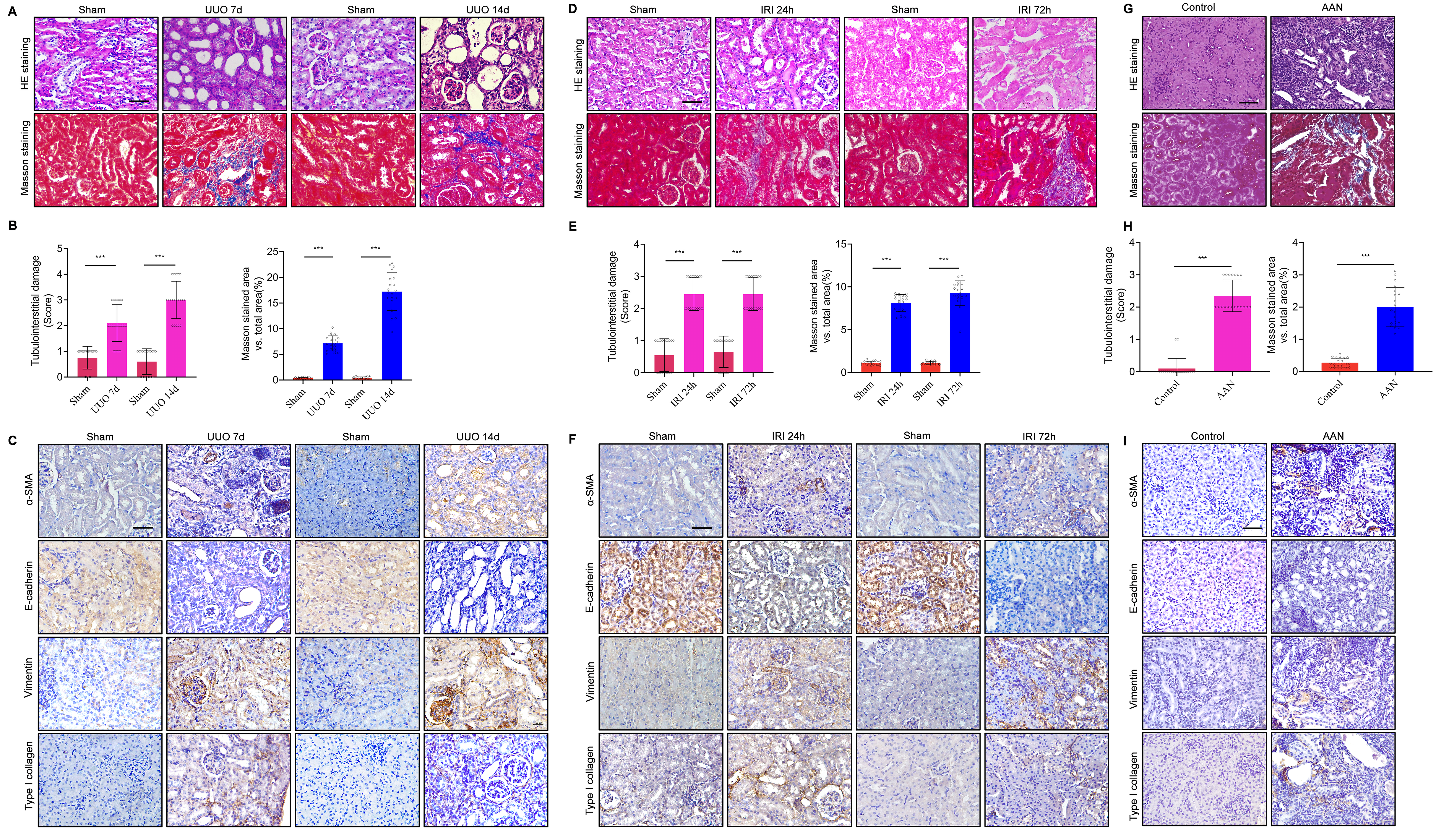

Supplement: Supplementary file 2 — High resolution image (TIF 42.6 mb) [file 10565_2024_9929_MOESM1_ESM.tif]

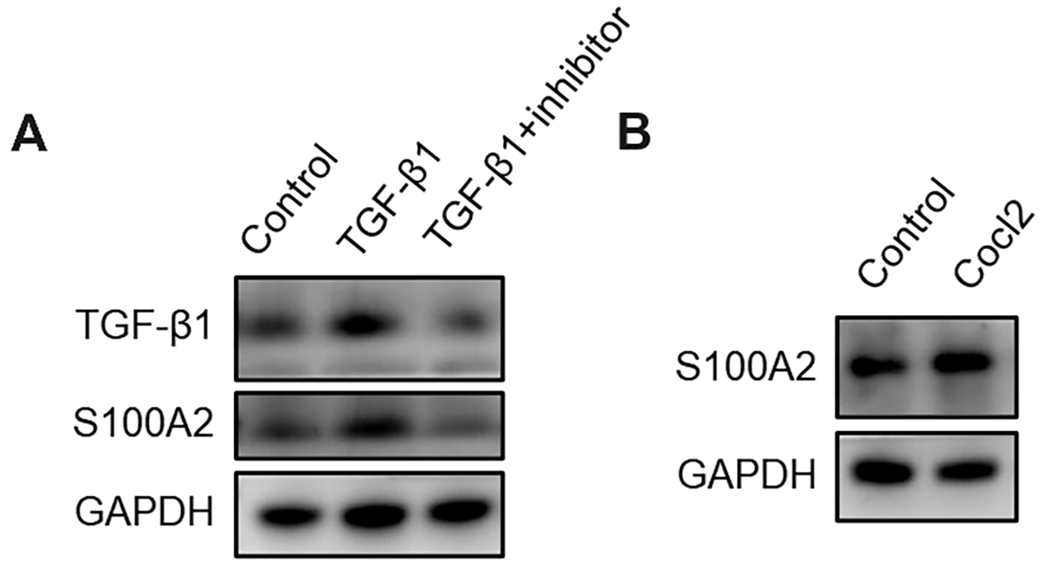

Supplement: Supplementary file 3 — Supplementary Fig. 2 Expression of S100A2 in HK2 cells treated with TGF-β1 inhibitor or CoCl2. (A) The TGF-β1 inhibitor antagonized the protein expression of S100A2. (B) CoCl2 induced high expression of S100A2. (PNG 116 kb) [file 10565_2024_9929_Fig8_ESM.png]

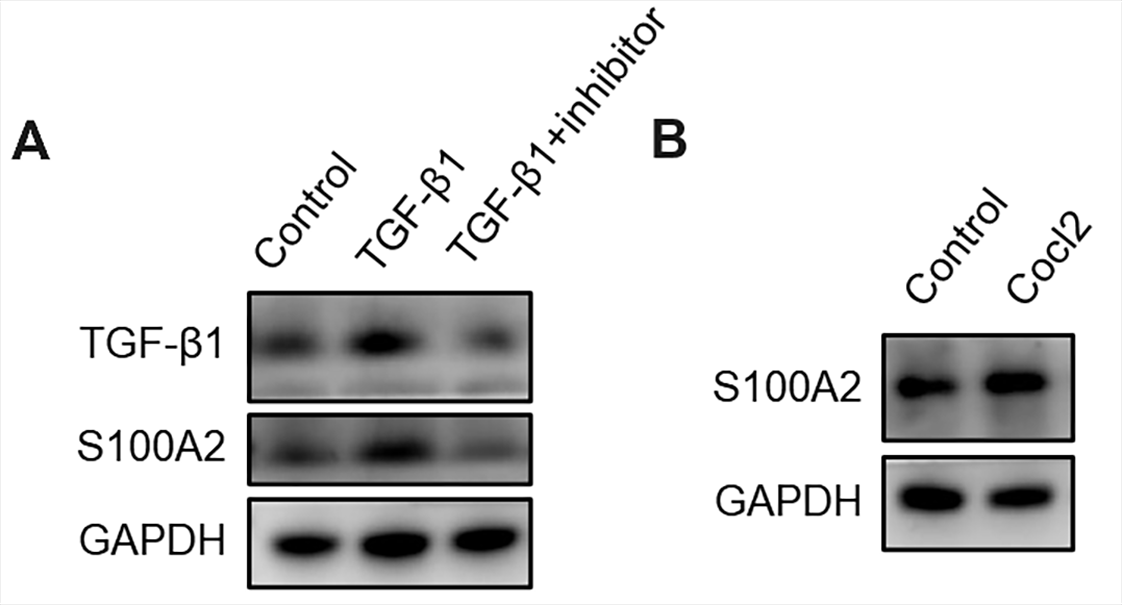

Supplement: Supplementary file 4 — High resolution image (TIF 186 kb) [file 10565_2024_9929_MOESM2_ESM.tif]

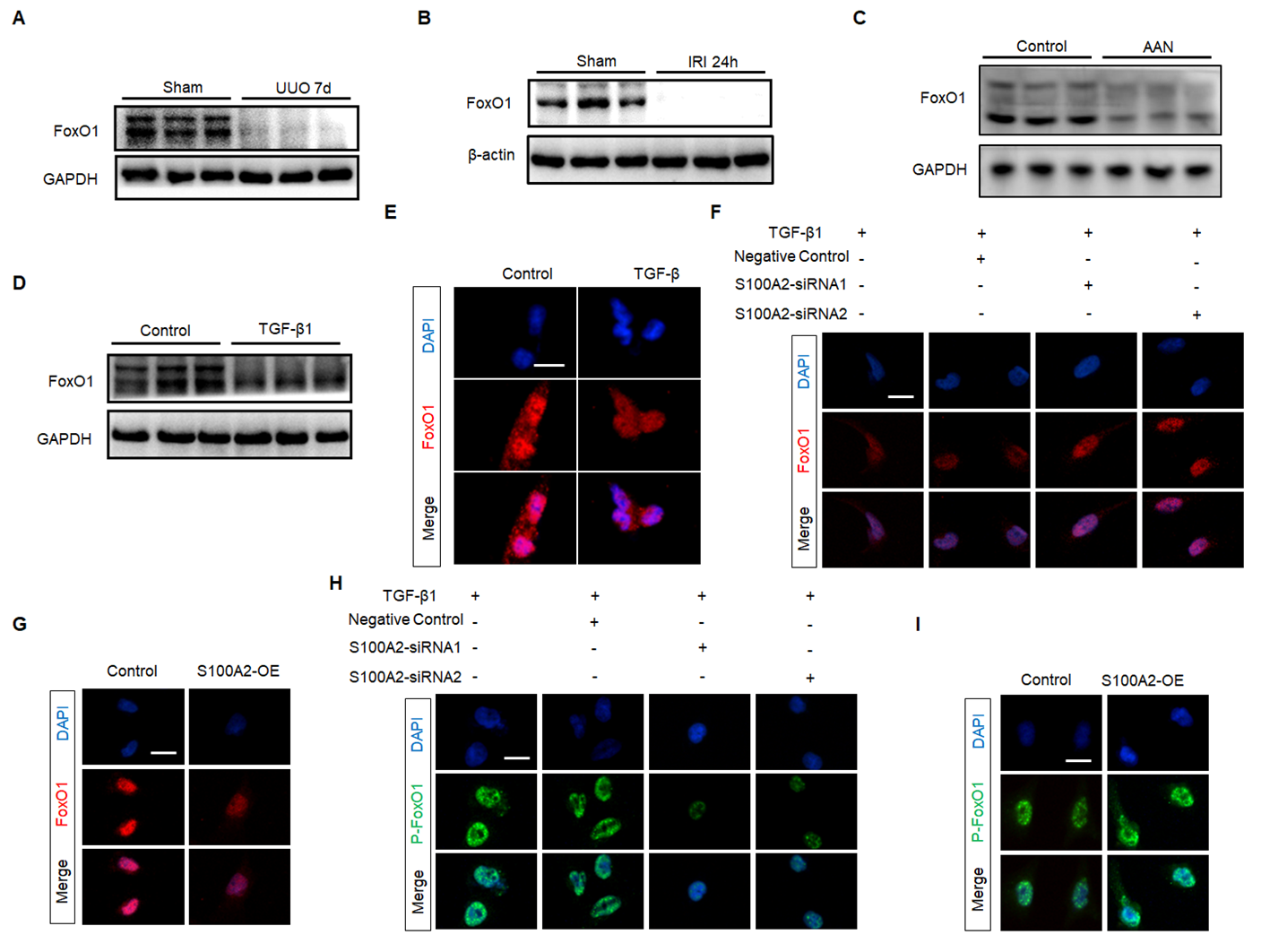

Supplement: Supplementary file 5 — Supplementary Fig. 3 Protein and subcellular localization of FoxO1 under different treatments. FoxO1 inhibition is induced by (A) UUO, (B) IRI, and (C) aristolochic acid. (D) Inhibition of FoxO1 protein expression and (E) nuclear localization in TGF-β1- stimulated HK-2 cells. Scale bar, 25 μm. (F) S100A2 knockdown enhances nuclear localization of FoxO1, while (G) S100A2 overexpression disrupts its nuclear expression. Scale bar, 25 μm. (H) S100A2 knockdown inhibits nuclear localization of p-FoxO1, while (I) S100A2 overexpression promotes its nuclear expression. Scale bar, 25 μm. (PNG 401 kb) [file 10565_2024_9929_Fig9_ESM.png]

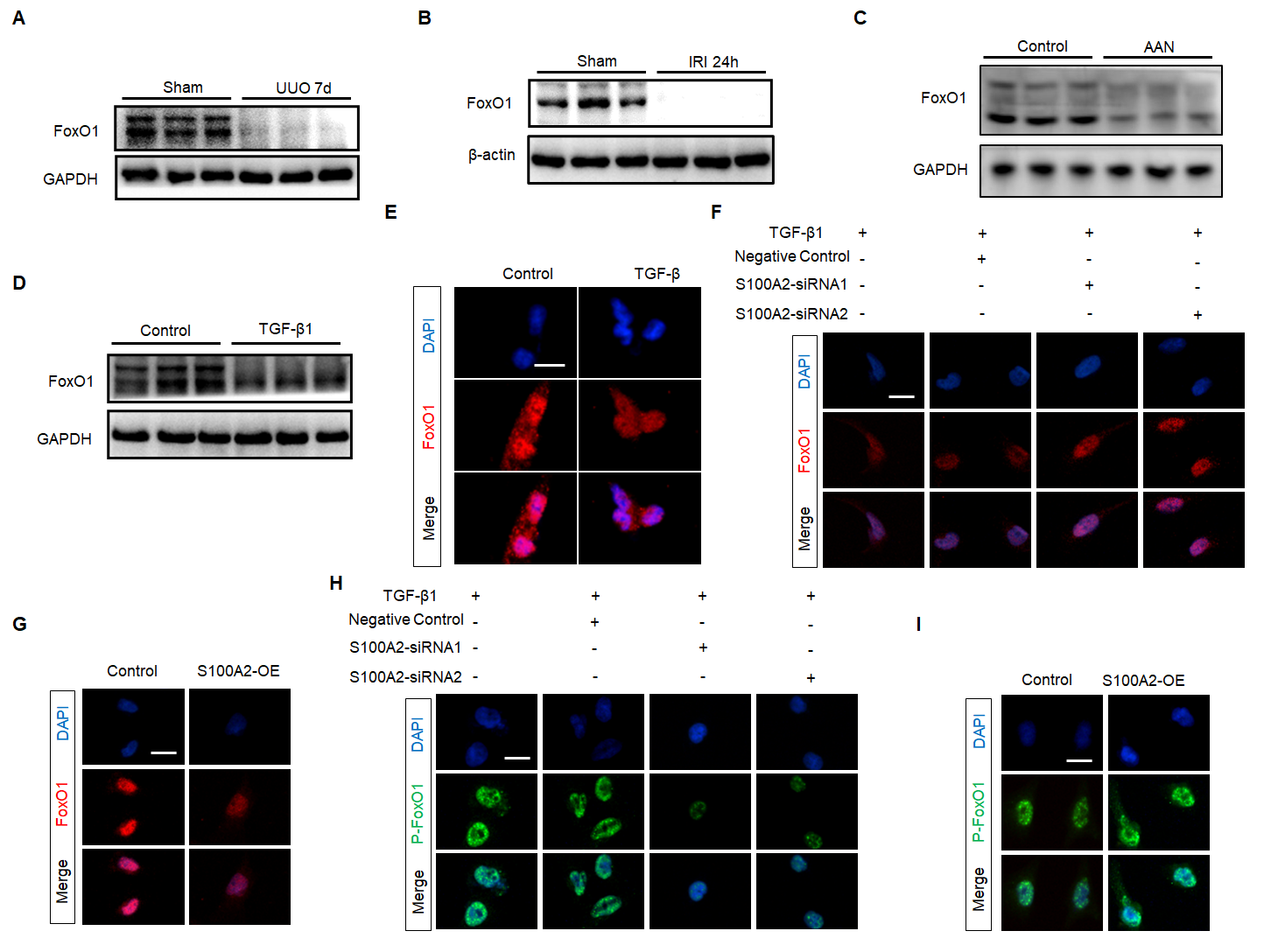

Supplement: Supplementary file 6 — High resolution image (TIF 941 kb) [file 10565_2024_9929_MOESM3_ESM.tif]
