# Supplementary material for: S100A2 activation promotes interstitial fibrosis in kidneys by FoxO1-mediated epithelial-mesenchymal transition
Source: Cell Biol Toxicol. 2024 Oct 9;40(1):86. doi: 10.1007/s10565-024-09929-7 (PMC11464619; doi:10.1007/s10565-024-09929-7)
Supplement: Supplementary file 7 — (DOCX 14 kb) [file 10565_2024_9929_MOESM4_ESM.docx]

Supplementary Table 1. PCR Primers used in this study

| **Gene** | **Sequence** |
| --- | --- |
| *F-Col1 (Mouse)* | GACAGGCGAACAAGGTGACAGAG |
| *R-Col1 (Mouse)* | CAGGAGAACCAGGAGAACCAGGAG |
| *F-α-SMA (Mouse)* | CGTGGCTATTCCTTCGTGACTACTG |
| *R-α-SMA (Mouse)* | CGTCAGGCAGTTCGTAGCTCTTC |
| *F-E-cadherin (Mouse)* | ATCCTGACCAGCAGTTCGTTGTTG |
| *R-E-cadherin (Mouse)* | GTTCCTCGTTCTCCACTCTCACATG |
| *F-S100A2 (Mouse)* | GAGGACGAGAGGCTCAAACACAAC |
| *R-S100A2 (Mouse)* | AGTCCACCTGCTGGTCACTGTC |
| *F-Vimentin (Human)* | AAGACACCATGAGCACAGAAAGC |
| *R-Vimentin (Human)* | AATCCTGCTCTCCTCGCCTTCC |
| *F-N-cadherin (Human)* | AGGAGTCAGTGAAGGAGTCAGCAG |
| *R-N-cadherin (Human)* | TTCTGGCAAGTTGATTGGAGGGATG |
| *F-E-cadherin (Human)* | GCCATCGCTTACACCATCCTCAG |
| *R-E-cadherin (Human)* | CTCTCTCGGTCCAGCCCAGTG |
| *F-Col3 (Human)* | CTCAGGGTGTCAAGGGTGAAAGTG |
| *R-Col3 (Human)* | TGTACCAGCCAGACCAGGAAGAC |
| *F-α-SMA (Human)* | CTTCGTTACTACTGCTGAGCGTGAG |
| *R-α-SMA (Human)* | CCCATCAGGCAACTCGTAACTCTTC |
| *F-* *S100A2 (Human)* | ATTCCAGAGAACCATGTGCTGTGAG |
| *R-* *S100A2 (Human)* | AGTGCTGTGTGATCTTGGGACAATC |
| *F-β-actin (Human)* | ACTGTCCACCTTCCAGCAGA |
| *R-β-actin (Human)* | AGCTCAGTAACAGTCCGCCT |
